# Transcriptional analysis of THP-1 cells infected with *Leishmania infantum* indicates no activation of the inflammasome platform

**DOI:** 10.1371/journal.pntd.0007949

**Published:** 2020-01-21

**Authors:** Mariana Gatto, Patrícia Aparecida Borim, Ivan Rodrigo Wolf, Taís Fukuta da Cruz, Gustavo Augusto Ferreira Mota, Aline Márcia Marques Braz, Bárbara Casella Amorim, Guilherme Targino Valente, Marjorie de Assis Golim, James Venturini, João Pessoa Araújo Junior, Alessandra Pontillo, Alexandrina Sartori

**Affiliations:** 1 Tropical Diseases Department, Botucatu Medical School – UNESP, Botucatu, Brazil; 2 Bioprocess and Biotechnology Department, Agronomic Sciences School – UNESP, Botucatu, Brazil; 3 Microbiology and Immunology Department, Biosciences Institute - UNESP, Botucatu, Brazil; 4 Internal Medicine Department, Botucatu Medical School – UNESP, Botucatu, Brazil; 5 Flow Cytometry Laboratory, Botucatu Medical School - UNESP, Botucatu, Brazil; 6 Medical School – UFMS, Campo Grande, Brazil; 7 Immunology Department, São Paulo University – USP, São Paulo, Brazil; Instituto de Ciências Biológicas, Universidade Federal de Minas Gerais, BRAZIL

## Abstract

Leishmaniasis is caused by intracellular parasites transmitted to vertebrates by sandfly bites. Clinical manifestations include cutaneous, mucosal or visceral involvement depending upon the host immune response and the parasite species. To assure their survival inside macrophages, these parasites developed a plethora of highly successful strategies to manipulate various immune system pathways. Considering that inflammasome activation is critical for the establishment of a protective immune response in many parasite infections, in this study we determined the transcriptome of THP-1 cells after infection with *L*. *infantum*, with a particular focus on the inflammasome components. To this end, the human cell line THP-1, previously differentiated into macrophages by PMA treatment, was infected with *L*. *infantum* promastigotes. Differentiated THP-1 cells were also stimulated with LPS to be used as a comparative parameter. The gene expression signature was determined 8 hours after by RNA-seq technique. Infected or uninfected THP-1 cells were stimulated with nigericin (NIG) to measure active caspase-1 and TNF-α, IL-6 and IL-1β levels in culture supernatants after 8, 24 and 48 hours. *L*. *infantum* triggered a gene expression pattern more similar to non-infected THP-1 cells and very distinct from LPS-stimulated cells. Some of the most up-regulated genes in *L*. *infantum*-infected cells were *CDC20*, *CSF1*, *RPS6KA1*, *CD36*, *DUSP2*, *DUSP5*, *DUSP7* and *TNFAIP3*. Some up-regulated GO terms in infected cells included cell coagulation, regulation of MAPK cascade, response to peptide hormone stimulus, negative regulation of transcription from RNA polymerase II promoter and nerve growth factor receptor signaling pathway. Infection was not able to induce the expression of genes associated with the inflammasome signaling pathway. This finding was confirmed by the absence of caspase-1 activation and IL-1β production after 8, 24 and 48 hours of infection. Our results indicate that *L*. *infantum* was unable to activate the inflammasomes during the initial interaction with THP-1 cells.

## Author summary

Visceral leishmaniasis, caused by *Leishmania infantum*, is a disease that affects millions of people worldwide. The entry of microorganisms into the host is commonly associated with activation of a multiprotein platform called inflammasome whose assembly culminates in caspase-1 activation and IL-1β production. ILβ activates other cells and effector mechanisms leading to clearance of pathogens. However, the involvement of inflammasomes in the human infection with *L*. *infantum* is poorly known. To investigate the parasite-host interaction is fundamental to understand the immunopathogenesis of visceral leishmaniasis and to allow the development of new therapeutic strategies. In this study, we used RNA-seq, a tool that allowed to investigate the global gene expression of THP-1 cells, which is a macrophage-like human cell line, infected with *L*. *infantum*. By using computational analysis, this approach allowed us to evaluate the expression of genes that compose the inflammasomes pathway and other gene networks and signaling pathways triggered after infection. This analysis indicated that, unlike species causing cutaneous leishmaniasis, *L*. *infantum* did not induce the expression of genes of inflammasome pathways, nor caspase-1 activation or IL-1β production, possibly reflecting a parasite strategy to manipulate immune system and therefore, to allow its survival inside the cells.

## Introduction

Leishmaniasis are a group of increasingly prevalent diseases transmitted to humans by sandflies, mainly *Phlebotomus* and *Lutzomya* [1]. Very distinct outcomes as cutaneous lesions, mucosal lesions and visceral involvement can occur depending upon the parasite specie and the immune condition of the vertebrate host [2]. Approximately 0.2 to 0.4 million visceral cases and 0.7 to 1.2 million cases of cutaneous leishmaniasis occur each year. More than 90% of visceral cases occur in six countries: India, Bangladesh, Sudan, South Sudan, Ethiopia and Brazil [3].

The complex immunopathogenesis of visceral leishmaniasis (VL) relies, among other aspects, on the variety of species and on the sophisticated way that they master the immunological response [2]. Macrophages play a pivotal role in these diseases; they are the primary resident cells and they are permissive for parasite proliferation [4]. However, they are also considered the most relevant effector cells being responsible for *Leishmania* elimination through activation of inflammatory signaling pathways and oxidative burst [5–7]. After the initial internalization of promastigotes by macrophages, there is the expected fusion of phagosomes and lysosomes [2,8]. However, *Leishmania* are among the few protozoa that are able not only to survive but also to multiply in this very inhospitable environment [9,10]. In order to do so, these parasites developed a plethora of highly successful strategies to manipulate the immune system [11]. The establishment of the infection therefore depends on the efficiency of the host to induce effector immune response and the parasite's efficiency to subvert the immune response of the host [11].

The production of pro-inflammatory cytokines as IL-1β and IL-18 by monocytes and macrophages at this early interaction will dictate much of the infection evolution [12]. Besides intensifying antimicrobial properties of phagocytes, these two cytokines are also involved in Th1 and Th17 polarization [13–15]. Differently from the majority of the cytokines, IL-1β and IL-18 are initially synthesized as inactive precursors that demand further cleavage, usually by intracellular caspases, to become biologically active [16,17]. The participation of cysteine protease caspase-1, the main enzyme involved in this process, depends upon the recruitment of a multiprotein platform termed inflammasome, that when stimulated is able to activate caspase-1 that cleaves pro-IL-β and pro-IL-18 into fully bioactive proteins [18,19].

The inflammasome is basically composed of a cytoplasmic sensor, an adapter protein ASC (apoptosis-associated speck-like protein) and caspase-1 [19]. The inflammasome-forming sensors (NLRP1, NLRP3, NLRP6, NLRP12, NLRC4) are pattern-recognition receptors (PRRs) belonging to the family of NLRs (nucleotide-binding and oligomerization domain like-receptors) or to HIN-200 family member as AIM2 [20]. The general structure of the NLRs members includes an N-terminal protein domain (pyrin domain, PYD or caspase recruitment domain, CARD), a central nucleotide binding and oligomerization domain (NAHCT) and a C-terminal domain (LRR, leucine-rich repeats). [21]. After ligand recognition, the sensor undergoes oligomerization and recruits the adaptor molecule ASC through PYD-PYD interactions, leading to activation of pro-caspase-1 into the caspase-1 enzymatically active which then cleaves pro- IL-18 and IL-1β [21].

The inflammasome assembly that takes place after the recognition of diverse pathogen-associated molecular patterns (PAMPs) and danger-associated molecular patterns (DAMPs) in the cytosol, occurs in two steps: *priming* signal which can be induced by Toll-like receptors (TLRs), NLRs and NF-κB activation which up-regulates transcription of inflammasome components such as pro-IL-1β, sensors and caspase-1 [21–23]. The second signal allows oligomerization of NLRs following the assembly of NLR, ASC and caspase-1 and consequent cleavage of pro-IL-1β and pro-IL-18 [24]. The second signals are mediated by host-derived molecules that include, for example, K^+^ efflux [25], extracellular ATP [26], mitochondrial dysfunction and reactive oxygen species (ROS) [27], lysosomal damage [28,29] and environment irritants such as silica and asbestos [30].

The proof-of-concept that inflammasome supports host resistance to *Leishmania* in mice genetically modified and murine cell lines infected with cutaneous species such as *L*. *amazonensis* and *L*. *major* was already demonstrated [31,32]. Such studies showed that these species trigger NLRP3 and caspase-1 activation and subsequent IL-1β release [31,32]. In contrast, NLRP3 inflammasome activation was not detected in human cells-infected with *L*. *major* [33]. Also, recent studies in mice infected with *L*. *donovani* and *L*. *guyanensis* showed that these parasites blocked the activation of inflammasomes [34,35]. Although the contribution of NLRs and inflammasome in the response to *Leishmania* has been studied, mainly in mice, its role during human visceral leishmaniasis is still unclear and needs to be further investigated.

A more recent methodological tool to investigate the details of this sophisticated interplay between *Leishmania* and the cells of the immune system has been the transcriptome profiling done by RNA-seq [36,37]. This approach allows an accurate and all-encompassing screening of gene expression of both, the parasite itself and the vertebrate host cell [37]. Concerning host cells, studies focusing in transcriptome profile have been done mainly with human macrophages infected with *L*. *major*, *L*. *amazonensis* and *L*. *panamensis* [37,38], U937-derived macrophages infected with *L*. *braziliensis* [39] and peripheral blood mononuclear cells from patients with visceral leishmaniasis [40]. However, studies concerning the global transcriptional profile with focus in the inflammasome platform from macrophages infected with *L*. *infantum* are not available yet.

In this context, we conducted a transcriptomic profiling of THP-1 cells infected with *L*. *infantum* to characterize the global gene expression and to investigate if this *Leishmania* specie activates the inflammasome platform. The effect on inflammasome was further analyzed by evaluating caspase-1 activation and IL-1β production.

## Materials and methods

### Ethics statement

A commercial cell line, human leukemia monocytic cell line (THP-1), obtained from the American Type Culture Collection (ATCC-TIB-202) was used in the study. The study was approved by the Research Ethics Committees of the Botucatu Medical School, under protocol 2.018.399.

### THP-1 culture and differentiation

THP-1 cells were cultured in 25 cm^2^ sterile culture flasks (Corning Incorporated, Corning, NY, EUA) in complete medium containing RPMI 1640 medium (Sigma-Aldrich, St. Louis, MO, EUA), supplemented with 10% heat-inactivated fetal bovine serum (Sigma) and 1% penicillin-streptomycin (Life Technologies, Carlsband, CA, EUA). The cells were maintained at the density of 10^6^ cells/mL and flasks with cell suspension were kept at 37°C and 5% CO_2_. The culture medium was exchanged every two days. THP-1 cells were plated in 24-well culture plate (TPP, Lab-Tek Chamber Slide System, USA) at a concentration of 5x10^5^cells/mL in complete medium, treated with 100 ng/mL of 4α-phorbol 12-myristate 13-acetate—PMA (Sigma-Aldrich) and incubated during 48 hours at 37°C and 5% CO_2_ prior to infection. PMA was used to induce differentiation of THP-1 cells into macrophage-like cells and to increase their phagocytic properties. Cell viability was evaluated before cell cultures by Trypan Blue. After stimulation with PMA, the cells displayed macrophage characteristics such as morphological alterations and adherence to culture plates.

### Parasites

The promastigote stage of *Leishmania infantum*, strain HU-USF 14, was cultured in Schneider´s *Drosophila* medium (Sigma) supplemented with 20% heat-inactived fetal bovine serum (Sigma), pH 7.0, 2% human urine, 5% Penicillin-Streptomycin (Sigma) and 5% L-glutamin (Life Technologies). *Leishmania* parasites were maintained at 25°C and sub-cultured every 3 days. Parasites were used for THP-1 cells infection after 5 days culture, that is, at the stationary phase. After eight culture passages, the parasites were serially passed through BABL/c mice to ensure the maintenance of their virulence.

### THP-1 infection and stimulation

Differentiated THP-1 cells (5 x 10^5^ cells/mL) were distributed in 24-well culture plates in complete medium and infected with *L*. *infantum* stationary phase promastigotes (10 parasites: 1 cell). Co-cultures were initially incubated during 4 hours at 37°C, 5% CO_2_ to allow parasite internalization. Next, non-ingested parasites were thoroughly washed off with RPMI-1640 medium and plates were incubated for additional 8, 24 or 48 hours in fresh complete medium at 37°C, 5% CO_2_. Under the same conditions, uninfected macrophages were stimulated with ultra-pure lipopolysaccharide—LPS (1 μg/mL) (InvivoGen, San Diego, CA, USA) and used as a positive control of cellular activation and expression of inflammasome proteins. In some experiments, infected and uninfected cells and LPS-stimulated cells were stimulated with nigericin (16 μM) (InvivoGen) for 1-hour, as the second signal for inflammasome activation. Uninfected non-stimulated cells were used as a negative control. The plates were incubated for 8, 24 and 48 hours at 37°C, 5% CO_2_.

After infection, cell viability was assessed by using Trypan Blue and experiments were conducted with cells whose viability was between 80–85%. The rate of infection was determined in chamber slides that were fixed and stained with Giemsa solution (LabHouse, Belo Horizonte, MG, Brazil) and number of intracellular parasites/100 macrophages was counted using light microscopy. For the assays, were used cultures with 70–80% infection rate and intracellular parasite number ranging 2–10 per cell (S1 File).

### Library preparations and sequencing

RNA extraction was performed and the global transcriptome profile of THP-1 cells was characterized by using RNA-seq technique. The experiments were performed in triplicate for uninfected, LPS-stimulated and *L*. *infantum*-infected macrophages. The cells were released from culture plates 8 hours after infection and RNA was extracted by using Total RNA Purification Kit (Norgen Biotek, Thorold, ON, Canada) following the manufacturer protocol. The total RNA was treated with DNAse kit (Qiagen, Valencia, USA). RNA quality and quantity were determined using a *NanoDrop 2000* spectrophotometer (Thermo Scientific, River Oaks Parkway, San Jose, CA) and all samples had a 260/280 ratio above 2.0. RNA integrity was evaluated using an *Agilent 2100 Bioanalyzer* system with an *RNA 6000 Nano Lab Chip* kit (Agilent Technologies, Santa Clara, EUA). All samples had an RNA integrity number (RIN) greater than 9.0. RNA samples were kept at -80°C until further processing.

Poly(a)-enriched cDNA libraries were generated using the *SureSelect Strand-Specific RNA Library Preparation Kit* for Illumina Multiplexed Sequencing (Agilent) and further purifications were performed using the *AMPure XP beads* kit (Beckmann Coulter, Fullerton, EUA). Next, cDNA libraries quantification was determined by RT q-PCR (7500 fast Real Time PCR Systems—Applied Biosystems) using *Kapa Library Quantification kit* (Kapa Biosystems, Boston, Massachusetts, EUA), following manufacturer protocols. The library product after *Kapa* quantification was 350 bp and the samples pool was sequenced using *Illumina NextSeq 500* (Illumina). The RNA-seq data is available at NCBI SRA under study number PRJNA552352.

### Bioinformatic analysis

The reads of each library were evaluated with the FastQC program [41] and cleaned with Trimmomatic v0.36 [42] with parameters “ILLUMINACLIP:./illumina_adapters.fa:4:30:10 LEADING:3 TRAILING:3 SLIDINGWINDOW:4:25 MINLEN:60”. The reads were then aligned with the HISAT [43] (default) against the *Leishmania infantum* genome (assembly accession GCA_000002875.2) available from the NCBI database [44] to exclude undesirable reads. The aligned reads were removed from the libraries and the remainder ones were mapped with the HISAT2 [43] (default) against *Homo sapiens* genome (GRCh38, gencode v24) available in the GENCODE database [45]. The analysis was then directed to evaluate differential expression, pathways and ontological terms enrichment analysis. Gene counts were generated with BEDTools intersect v2.25.0 [46] (default), based on the annotations provided with the human genome. The differential gene expression was performed by DESeq2 [47] version 1.14.1 [48] in which the p-values were adjusted for multiple testing using the procedure of Benjamini and Hochberg. In summary, the DESeq2 compares replicates of samples and normalizes the data by Rlog. Then, the Wald test was used to evaluate the significance of the expression followed by adjustment using Benjamini and Hochberg procedure. Genes with False Discovery Rate (FDR) were considered differentially expressed and separated into up or down- regulated based on the *Log2 fold-change* provided by DESeq2. The enrichment analysis for ontological terms (GOs) annotations [49] and for pathways present in the KEGG database [50] were performed by using the GAGE R package [51]. Enriched pathways visualizations were obtained by the Pathview R package [52]. The enrichment analysis allowed the identification of biological identities associated with differentially expressed genes. The GOs and pathways with FDR <0.01 were considered significant.

### Caspase-1 active assay

To assess the activity of caspase-1 was used the FAM-FLICA *in vitro* Caspase Detection Kit (Immunochemistry, Bloomington, MN, USA), following the manufacturer’s instructions. Caspase-1 activity was evaluated in THP-1 cells infected with *L*. *infantum* and in cells stimulated with LPS. Cells that were neither infected nor stimulated were used as control. After 8, 24 and 48 hours of incubation, THP-1 cells were removed from the wells by further incubation in phosphate buffer saline (PBS) containing 0,5 mM of EDTA, at 37°C for 20 minutes. The cells were harvested, centrifuged and then incubated with FAM-FLICA during 1 hour at 37°C, 5% CO_2_. The cells were then washed with wash buffer, centrifuged and then resuspended. Analysis and cell acquisition were performed via flow cytometry (FACSCalibur^™^, Becton, Dickinson and Company, Franklin Lakes, NJ, USA) using the Cell Quest software (Becton, Dickinson and Company). Acquisition was standardized to 20.000 events per sample.

### Cytokine production

IL-1β, TNF-α and IL-6 were measured in culture supernatants from THP-1 cells infected with *L*. *infantum* or stimulated with LPS during 8, 24 and 48 hours. Infected and non-infected THP-1 cells were also stimulated with nigericin. These evaluations were done by ELISA kit (R&D Systems, Minneapolis, MN, USA), according to the manufacturer’s instructions.

### Statistical analysis of cytokines and caspase-1

Analysis of variance (ANOVA) for repeated measurements followed by the Tukey test was used when comparing the groups. The differences were considered significant for p<0.05. Statistical analysis was performed using GraphPad Prism 5.00 software (San Diego, CA, USA).

## Results

### Global transcriptional profiling of THP-1 cells infected with *L*. *infantum*

**Fig 1:**
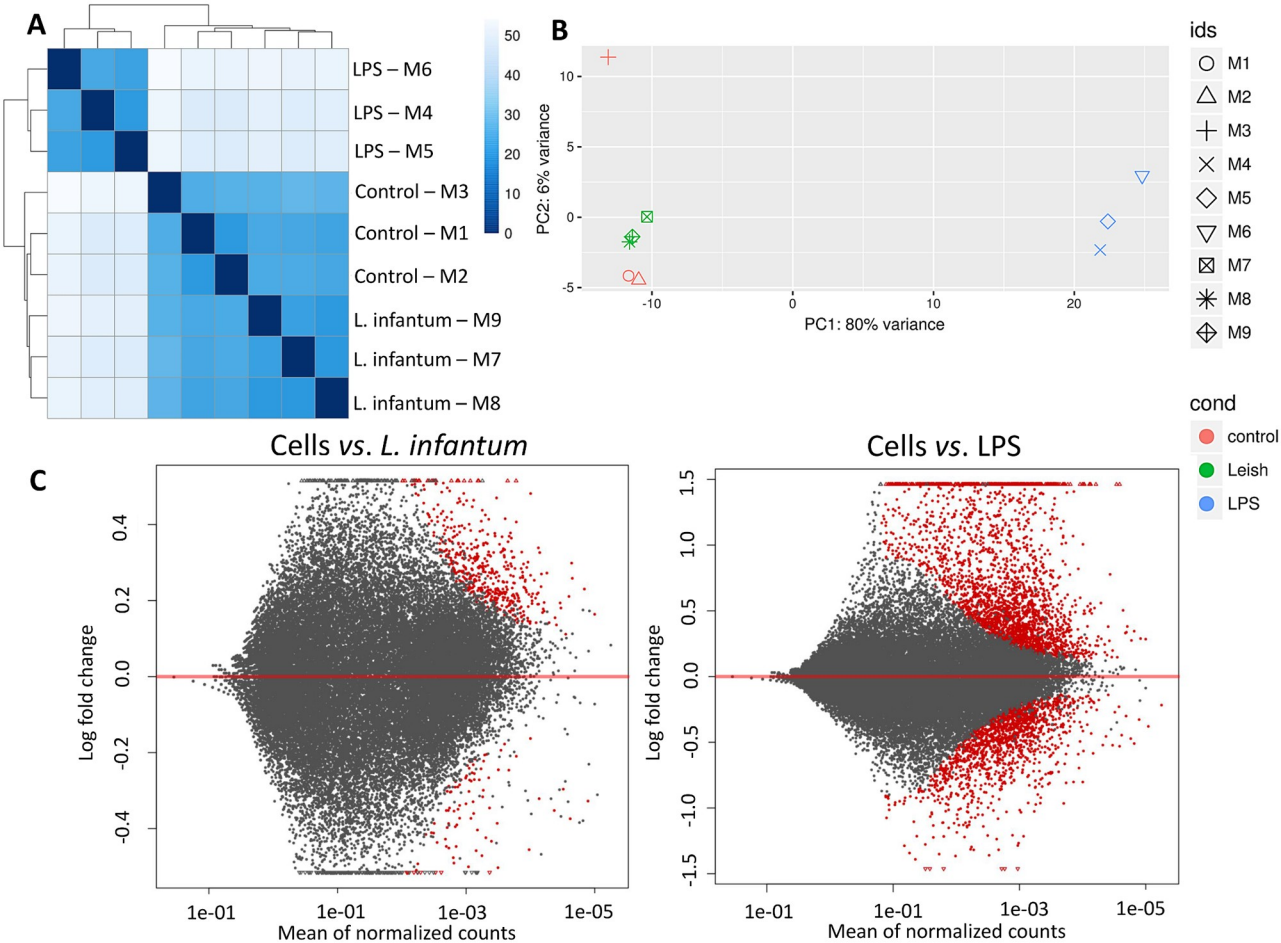
Global transcriptional expression patterns from *L*. *infantum*-infected cells or LPS-stimulated cells. **A)** The heat map was performed by using the Euclidean distance method with complete linkage for all samples (M1, M2, M3 are triplicates of non-stimulated cells; M4, M5, M6 are triplicates of LPS-stimulated cells; M7, M8, M9 are triplicates of *L*. *infantum*-infected cells). **B)** The principal component analysis (PCA) was performed with normalized data: PC1 (axis x) shows the variance (%) among the groups (non-stimulated, LPS-stimulated and infected cells) and PC2 (axis y) shows the variance (%) among the samples. **C)** The MA-plot shows the distribution of the gene expression between *L*. *infantum*-infected cells or LPS-stimulated cells and non-stimulated cells. The axis X shows the mean of normalized counts and axis Y shows the comparison by Log2fold change (Log2FC). Red dots correspond to genes up-regulated (above 0) and down-regulated genes (bellow 0) based on the False Discovery Rate (FDR<0.01).

The human THP-1 cell line is being widely employed to study different aspects of the interaction between *Leishmania spp*. and the vertebrate host cells [5,53]. In this sense, after differentiation into macrophages by PMA, THP-1 cells were used to characterize their global gene expression in response to *L*. *infantum* infection and to determine if this parasite was able to up-regulate the expression of genes related to NLRs and inflammasomes. This transcriptional analysis was performed in three conditions: cells, cells infected with promastigotes of *L*. *infantum* and cells stimulated with LPS, a well-recognized stimulus for *in vitro* models of inflammation [17,54,55], to be used as a comparative parameter. All three conditions were run in triplicates: M1, M2 and M3 for non-stimulated cells, M4, M5 and M6 for cells stimulated with LPS and M7, M8 and M9 for cells infected with *L. infantum*. The global transcript profiles were evaluated 8 hours after incubation by the RNA-seq technique, being this time period rationally chosen to allow the characterization of the transcriptional profile at an early stage of infection. The RNA-seq generated approximately >9 million reads per sample, ranging from 60–76 nts. After exclusion of reads from *L*. *infantum* genome, the remaining reads were mapped on the human genome (S2 File) allowing mapping of 45,439 genes. After data normalization by R-log, the libraries were analyzed by Euclidean distance algorithm and by PCA (*Principal Component Analysis*). The heatmap displayed in Fig 1A shows desirable distances and similarities among the libraries, revealing that all samples were correctly grouped into two distinct clusters. The cluster constituted by the genes expressed by both, non-stimulated and *L*. *infantum*-infected cells, showed that gene expression patterns in these two groups were similar; and the other cluster, formed by the genes expressed by LPS stimulated cells, indicated that this group was less similar to infected or non-stimulated cells (Fig 1A). The PCA (Fig 1B) showed that 80% of the variation in our data is explained by LPS treatment; in this case, the variance between infected cells and non-infected cells is low (PC1). Moreover, the samples did not present high variances among them (PC2) (Fig 1B).

Genes with FDR<0.01 were considered differentially expressed and based on the values of *Log2 fold-change* we identified which ones were up or down-regulated. According to MA-plot (Fig 1C), LPS stimulation triggered a striking alteration in the pattern of gene expression: 2,640 genes were up-regulated (*Log*_*2*_ *fold-change* from 0.11 to 6.15) whereas 1,583 were down-regulated (*Log*_*2*_ *fold-change* from -0.13 to -2.09) in comparison to non-stimulated THP-1 cells. On the other hand, infected THP-1 cells showed only 498 up-regulated genes (*Log2 fold-change* from 0.11 to 1.09) and 92 down-regulated genes (*Log2 fold-change* from -0.16 to -0.76) after 8 hours of infection (Fig 1C).

### The top 30 genes most significantly up and down regulated in THP-1 cells infected with *L*. *infantum*

**Fig 2:**
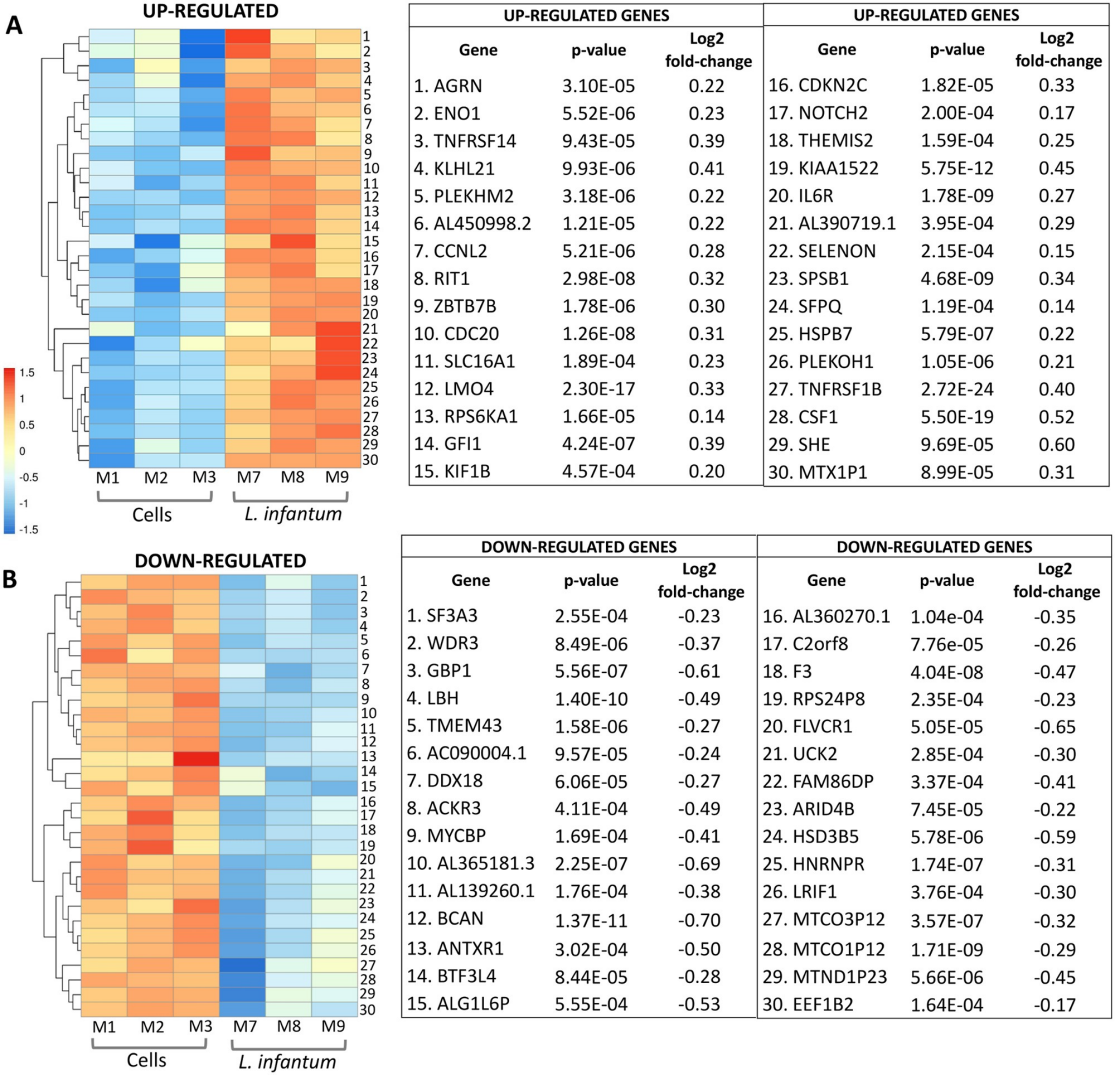
The top 30 genes most significantly up and down expressed by THP-1 cells infected with *L*. *infantum*. The heatmap shows the 30 most significantly up-regulated genes in *L*. *infantum*-infected cells (**A**) and the 30 most significantly down-regulated genes in *L*. *infantum*-infected cells (**B**) in comparison to non-stimulated cells after 8 hours incubation. The matrix was made by DESeq2 based on the *Log2 fold-change* (FC>0).

The 30 most significantly up or down-regulated genes in infected cells in comparison to non-stimulated cells were identified by DESeq2 package based on the *Log2 fold-change* (FC>0). A heatmap illustrates the 30 up-regulated genes (Fig 2A) and down-regulated genes (Fig 2B) in THP-1 infected cells. By using Reactome Pathway Database [56] and Kegg Database [50] were identified the functional pathways to which these genes were linked. The analysis identified up-regulated genes related to: cell cycle (*CDC20*, *CDKN2C*, *CNNL2*), glycolysis/gluconeogenesis (*ENO1*), metabolism of carbohydrates (*AGRN)*, signal transduction *(RIT1*, *RPS6KA1*, *ILR6*, *SFPQ)*, MAPK signaling *(RPS6KA1)*, gene expression (*NOTCH2*), ubiquitin mediated proteolysis (*CDC20*) and interleukin signaling (*TNFRSF14*, *TNFRSF1B*, *ILR6*, *CSF1*) (Fig 2A). Concerning the 30 genes down-regulated in THP-1 infected cells compared to non-stimulated cells, were found genes associated to pathways signaling such as: gene expression (*ARID4B*, *MYCBP*), metabolism of sucrose and folate (*DDX18*), signal transduction (*ACKR3*, *MYCBP*), interleukin signaling (*GBP1*), extracellular matrix organization (*BCAN*) and coagulation (*F3*) (Fig 2B).

### Functional enrichment pathway analysis of the THP-1 cell infected with *L*. *infantum*

**Fig 3:**
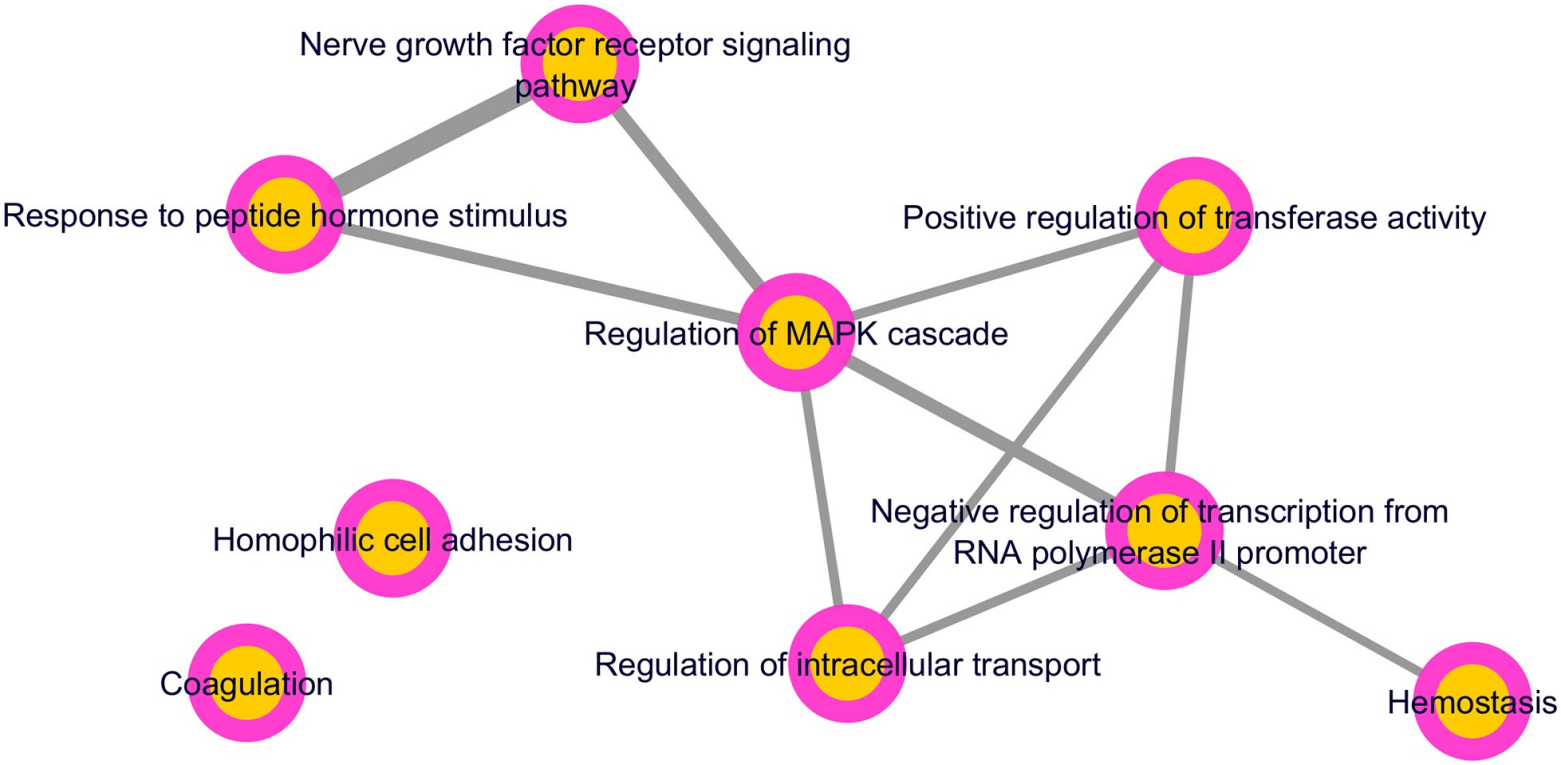
Functional enrichment analysis of *L*. *infantum*-infected cells in relation to unstimulated cells. Gene Ontology (GO) enrichment analysis summarized by REVIGO showing 9 biological process enriched (*parents* GO terms) in up-regulated genes (FDR<0.01) in *L*. *infantum*-infected cells after 8 hours. Highly similar GO terms are linked by edges, where the line width indicates the degree of similarity.

We next carried out a functional enrichment analysis using Gene Ontology (GO) terms by adopting the R GAGE package [51]. This allowed identification of biological entities linked to the differentially expressed genes by THP-1 cells infected with *L*. *infantum* or stimulated with LPS in comparison to non-stimulated cells. The R GAGE analysis identified the GOs categories significantly over-expressed which were summarized and redundant terms were removed. THP-1 cells stimulated with LPS presented 217 enriched biological processes up-regulated which were summarized in 40 *parents* GO terms (S3 File). In the other hand, *L*. *infantum* infection resulted in only 37 enriched biological processes linked to up-regulated genes, both in comparison to non-stimulated cells (FDR<0.01). Considering the 37 biological entities up regulated in *L*. *infantum* infection, 12 were also enriched in cells stimulated with LPS whereas the other 25 were found enriched exclusively in infected cells (9 *parents* GO terms and 16 *child* GO terms) (Table 1). The 9 *parents* GO terms up-regulated after 8 hours of *L*. *infantum* infection in THP-1 cells were associated to: cell adhesion (*ACTG1*, *ACTB*, *CTNNA1*, *PIK3R2*), coagulation (*CD36*, *FERMT3*, *CD109*, *PIK3R2*), regulation of MAPK cascade (*DUSP2*, *DUSP5*, *IL6R*, *IRS2*, *DUSP7*, *MAP3K1*), response to peptide hormone stimulus (*CTSZ*, *CTSD)*, negative regulation of transcription from RNA polymerase II promoter (*RGCC*, *ARID3A*, *CCND1*, *SGK1*, *IRAK1)*, regulation of intracellular transport (*MMP9*, *ANXA1*, *HMOX1*, *IER3*, *NEDD4L*, *IRAK1*, *IRS2*, *LGALS3)*, hemostasis (*SLC16A3*, *TUBB4B*, *CD36*, *FERMT3*, *CD109*), positive regulation of transferase activity (*ACTG1*, *ACTB*) and nerve growth factor receptor signaling pathway (*DUSP7*, *RPS6KA1*, *PIK3R2*, *IRS2*, *DNM2*, *RIT1*). The interactive graph of the 9 biological processes up-regulated in infected cells was represented by using REVIGO [57] and software Cytoscape [58] and is displayed at Fig 3.

**Table 1:** *Parents* and *child* GO terms enriched in up-regulated genes in *L*. *infantum*-infected cells in relation to non-stimulated cells.

Biological process	p-value	GO
**Response to peptide hormone stimulus**	**2.23E+09**	**GO:0043434**
*Cellular response to nitrogen compound*	7.81E+08	GO:1901699
*Cellular response to organic nitrogen*	5.44E+09	GO:0071417
*Cellular response to hormone stimulus*	9.25E+08	GO:0032870
*Response to peptide hormone*	1.20E+09	GO:1901652
**Positive regulation of transferase activity**	**2.40E+09**	**GO:0051347**
*Positive regulation of protein kinase activity*	5.11E+09	GO:0045860
*Positive regulation of protein serine/threonine kinase activity*	3.92E+08	GO:0071902
*Regulation of protein serine/threonine kinase activity*	1.03E+09	GO:0071900
*Positive regulation of kinase activity*	4.69E+09	GO:0033674
**Negative regulation of transcription from RNA polymerase II promoter**	**3.36E+09**	**GO:0000122**
*Negative regulation of cellular protein metabolic process*	5.56E+09	GO:0032269
*Negative regulation of protein metabolic process*	7.87E+08	GO:0051248
**Regulation of intracellular transport**	**4.59E+09**	**GO:0032386**
*Regulation of secretion*	6.71E+09	GO:0051046
*Negative regulation of transport*	3.70E+08	GO:0051051
**Hemostasis**	**4.68E+09**	**GO:0007599**
*Blood coagulation*	7.50E+09	GO:0007596
*Platelet activation*	9.79E+08	GO:0030168
**Coagulation**	**5.65E+09**	**GO:0050817**
**Regulation of MAPK cascade**	**1.21E+08**	**GO:0043408**
*Positive regulation of MAPK cascade*	3.81E+09	GO:0043410
**Nerve growth factor receptor signaling pathway**	**2.16E+08**	**GO:0048011**
**Homophilic cell adhesion**	**7.04E+08**	**GO:0007156**
*Cell-cell adhesion*	1.55E+09	GO:0016337

### Inflammasome genes expression is not induced by infection of THP-1 cells with *L*. *infantum*

One of the objectives of this study was to investigate if the expression of NLRs and inflammasome genes were modulated at the early stages of infection of THP-1 cells with *L*. *infantum*. The enrichment analysis for *Nod-like receptors signaling pathway* present in the KEGG database [50] was performed by the R GAGE package [51] and enriched pathways visualization was obtained by the Pathview R package [52]. Of the 170 genes composing the NLR pathway, only 9 were differentially expressed after infection with *L*. *infantum* (Table 2) and therefore this pathway was not significantly enriched by infection of THP-1 cells with *L*. *infantum*. This result shows that the parasite was not to activate inflammasomes at the initial stages of infection. On the other hand, the NLRs signaling pathway was significantly enriched in up-regulated genes in LPS-stimulated cells (FDR = 0.0006) compared to non-stimulated cells (S4 File). The Table 3 lists some of the most relevant up-regulated genes of the NLR signaling pathway in LPS-stimulated cells.

**Table 2:** Up and down-regulated genes related to NLRs pathway (KEGG) in THP-1 cells infected with *L*. *infantum* in relation to non-stimulated cells.

**Up-regulated genes**		
**Gene**	***p*-value**	**FC (*fold-change*)**
JUN/AP-1	9.47E+06	0.27
TNFAIP3/A20	5.15E+10	0.35
IRF7	7.44E+17	0.56
ANTXR2	1.24E+05	0.21
CTSB	2.88E+20	0.32
PRKCD/PKC	4.18E+06	0.2
**Down-regulated genes**		
**Gene**	***p*-value**	**FC (*fold-change*)**
CCL2/MCP-1	1.27E+05	-0.5
ANTXR1	3.02E+04	-0.5
GBP1	3.94E+05	-0.61

**Table 3:** Up-regulated genes related to NLRs pathway (KEGG) in LPS-stimulated THP-1 cells in relation to non-stimulated THP-1 cells.

Up-regulated genes		
Gene	*p*-value	FC (fold-change)
AIM2	4.97E+70	3.3
ANTXR2	1.81E+37	0,62
BIRC3/CIAP	4.38E+65	1.99
CARD6	4.27E+18	1.65
CASP1	8.97E+66	2.16
CCL2/MCP-1	0	3.73
CTSB	3.41E+05	0.14
GBP1	0	4.25
GBP2	0	4.81
GPB3	3.49E+245	3.01
GBP4	0	5.59
GBP5	0	4.84
GSDMD	3.93E+43	1.32
IFI16	1.69E+275	1.97
IKB/NEMO	9.80E+05	0.24
IL-1β	0	1.86
ITPR3	9.14E+09	0.8
IRF7	9.48E+264	2.17
IRF9	5.31E+93	1.32
JUN	1.19E+51	0.8
MEFV/PYRIN	2.18E+23	1.5
MYD88	6.31E+155	1.68
NFκB	1.29E+04	0.23
NLRP3	1.95E+54	0.97
NOD1	3.68E+04	0.68
NOD2	2.01E+24	2.21
OAS1	0	2.84
OAS2	0	3.14
OAS3	0	2.45
P2RX7	5.68E+78	2.89
PKC	2.27E+15	0.34
RBCK1	1.83E+47	1.03
RIPK2	5.73E+49	1.15
STAT1	0	2.28
STAT2	1.39E+259	2.35
TLR4	3.84E+13	0.58
TNFAIP3/A20	1.01E+268	1.86
TNF-α	8.39E+96	1.89
TRIF	1.15E+18	0.67

### Caspase-1 activation and IL-1β production are not triggered by infection of THP-1 cells with *L*. *infantum*

**Fig 4:**
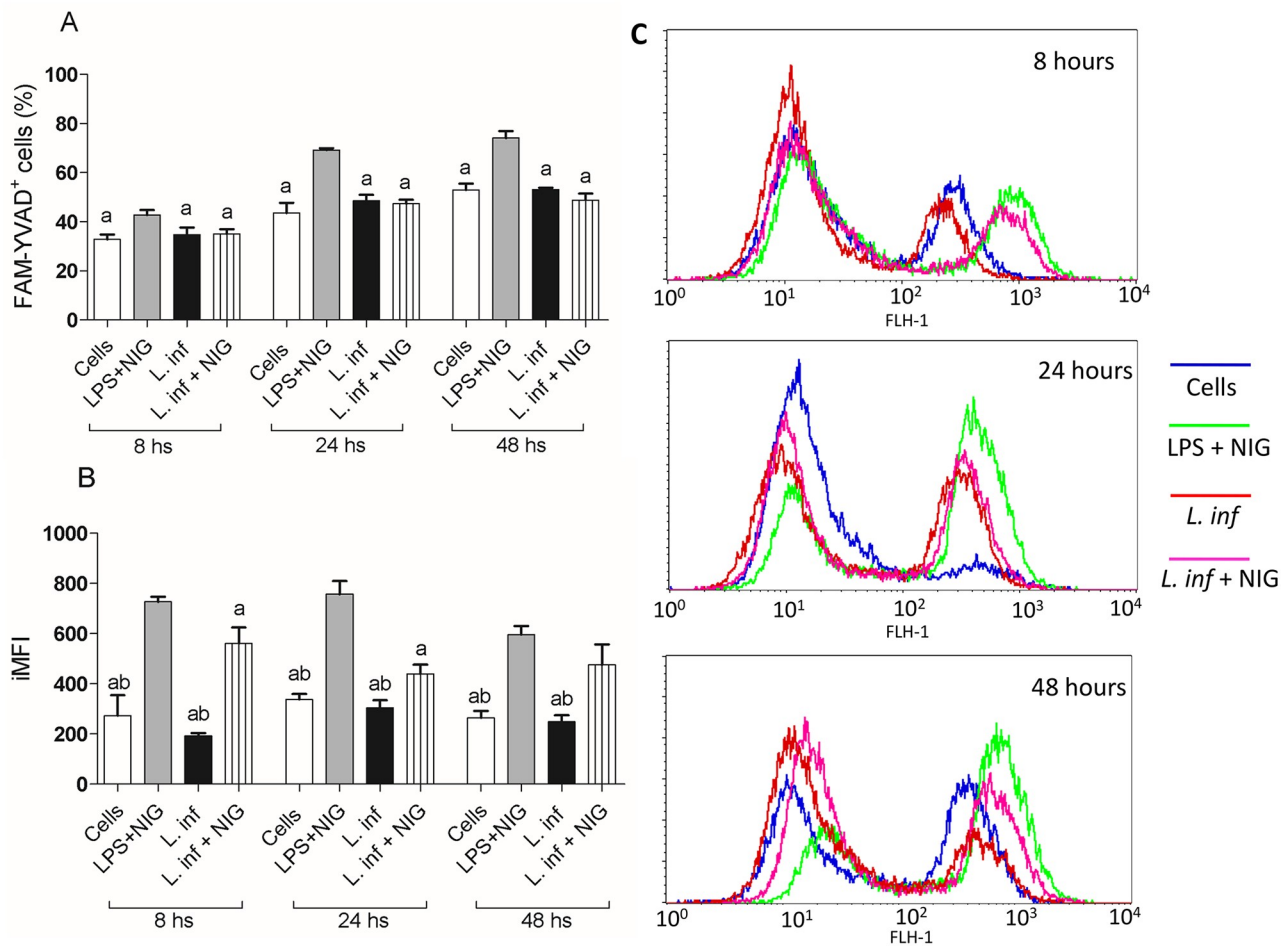
Active caspase-1 in THP-1 cells infected with *L*. *infantum*. THP-1 cells previously differentiated into macrophages were infected with *L*. *infantum* (1:10) and the percentage of cells expressing active caspase-1 **(A)**, the mean fluorescence intensity (MFI) **(B)** and representative histograms plots **(C)** were evaluated 8, 24 and 48 hours after infection. In addition to *L*. *infantum*, were also tested other stimuli as LPS + nigericin (LPS + NIG) and *L*. *infantum* + nigericin (L.inf. + NIG). Cultures were stimulated with nigericin during 1 hour before the endpoint of the tests. The results are representative of three independent experiments performed in triplicate. Results are expressed as mean ± SD: **a** *vs*. LPS+NIG, **b** *vs*. L. inf + NIG, p<0.05.

According to our transcriptional evaluation, *L*. *infantum* did not induce the expression of inflammasome genes in THP-1 cells after 8 hours of infection. Functional assays to detect activation of caspase-1 and IL-1β production were then performed to confirm these results. The evaluation period was extended, including 8, 24 and 48 hours. As expected, a higher percentage of THP-1 cells expressing active caspase-1 was observed in cultures stimulated with LPS plus nigericin (LPS+NIG) in comparison to non-stimulated cells, infected cells or infected cells plus nigericin (p<0.05), as demonstrated in Fig 4A. On the contrary, infection of THP-1 cells with *L*. *infantum* in the absence or presence of nigericin did not increase the percentage of cells expressing active caspase-1 in comparison to control non-infected/non-stimulated cells (Fig 4A). LPS+NIG treatment also significantly increased the mean fluorescence intensity of caspase-1 in comparison to non-stimulated cells (p<0.01), while THP-1 cells infected with *L*. *infantum* showed active caspase-1 levels similar to those found in non-stimulated cells. However, the mean fluorescence intensity significantly increased in *L*. *infantum*-infected cells when nigericin was also added (p<0.05), as can be observed at Fig 4B.

**Fig 5:**
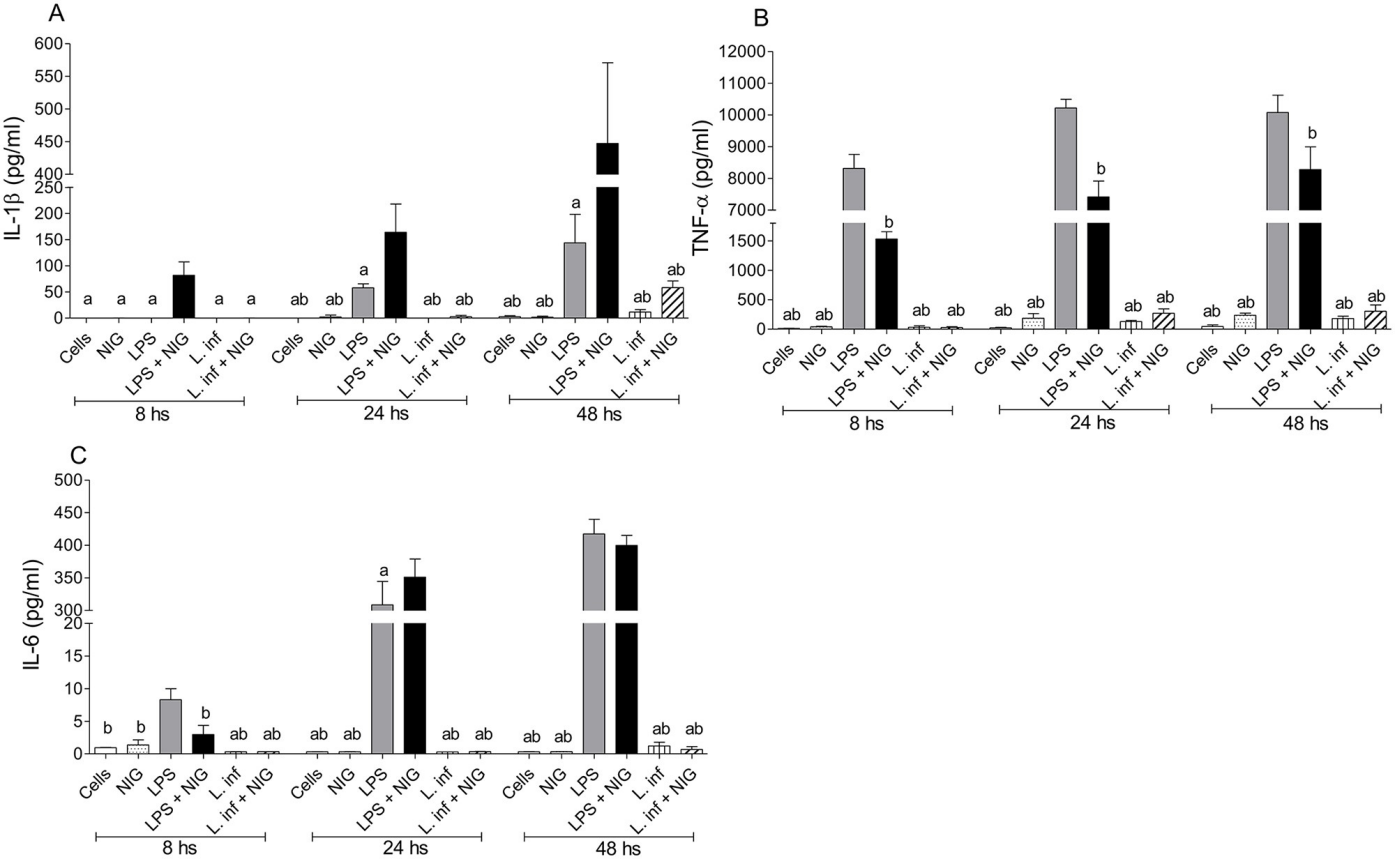
Production of cytokines by THP-1 cells infected with *L*. *infantum*. THP-1 cells previously differentiated into macrophages were infected with *L*. *infantum* (1:10) and levels of IL-1β **(A)**, TNF-α **(B)** and IL-6 **(C)** were evaluated in the supernatants of the cultures after 8, 24 and 48 hours of incubation. In addition to *L*. *infantum*, the following stimuli were tested: LPS, nigericin (NIG), LPS + nigericin (LPS + NIG) and *L*. *infantum* + nigericin (L. inf + NIG). Cultures were stimulated with nigericin during 1 hour before the endpoint of the tests. The results are representative of three independent experiments performed in triplicate. Data are expressed as mean ± SD: p <0.05 **a** *vs*. LPS + NIG, **b** *vs*. LPS.

To investigate the ability of *L*. *infantum* to induce the production of IL-1β (dependent upon inflammasome activation) and other pro-inflammatory cytokines as TNF-α and IL-6, that are independent of inflammasome activation, THP-1 cells were infected and cytokine levels were evaluated 8, 24 and 48 hours after. As already anticipated, LPS was able to induce IL-1β and nigericin addition to LPS stimulated cells promoted higher production of this cytokine. *L*. *infantum* by itself or together with nigericin was not able to induce significant amounts of IL-1β (Fig 5A). Very low levels of this cytokine were found, however, in cultures of THP-1 infected with *L*. *infantum* in the presence of nigericin, after 48 hours of incubation (Fig 5A). LPS and LPS + nigericin stimulation also induced significant levels of TNF-α and IL-6 in all evaluated time periods. These cytokines were not, however, detected in *L*. *infantum* infected cultures in any of the three analyzed time points (Fig 5B and 5C).

## Discussion

This study was designed to characterize the global gene expression in THP-1 cells infected with *L*. *infantum*; an emphasis was given to inflammasome activation because this platform is a pivotal and controversial subject in *Leishmania* infection. To this end, the human monocytic cell line THP-1 differentiated into macrophages was infected with *L*. *infantum* promastigotes. To capture the global transcriptional response during early stages of the infection, RNA-seq technique was performed 8 hours after *L*. *infantum* challenge.

The initial analysis revealed that non-stimulated and *L*. *infantum*-infected cells were clustered into a group whereas LPS-stimulated cells were clustered in a distinct group, already indicating that infected and control cells displayed a similar gene expression pattern. The analysis of differentially expressed genes reinforced these findings by showing that cells stimulated with LPS exhibited greater variation in gene expression than infected THP-1 cells in relation to non-stimulated cells. Corroborating these results, studies showed that human macrophages infected with *L*. *chagasi*, *L*. *mexicana* and *L*. *donovani* presented genetic patterns similar to the non-infected cells or suppression of gene expression in macrophages [4,59,60]. Contrastingly, infection with *L*. *panamensis*, *L*. *major* and *L*. *amazonensis* clearly induced a wider and more remodeling gene expression in human macrophages [37,38]. These divergent results suggest that the pattern of gene expression in macrophages depends, at least partially, on the parasite species involved in the infection.

Infection of macrophages with *Leishmania*s is complex and involves both, the ability of the host to activate effector mechanisms against the parasite and the parasite’s ability to suppress the host immune response [5]. Current reviews indicate that *Leishmania* species are among the parasites that developed very specialized strategies for immune evasion and survival [2,11]. The analysis of our results indicated that CDC20 and CSF1 genes were among the 30 most up-regulated genes in *L*. *infantum*-infected cells, being possibly correlated with parasite survival. The CSF1 gene encodes M-CSF which is an important cytokine involved in macrophage function and differentiation [61]. M-CSF is also associated with anti-inflammatory M2 macrophage polarization [62,63] what could allow parasite survival through an anti-inflammatory macrophage differentiation. Expression of CDC20 which is a crucial protein involved at multiple steps during cell cycle [64,65], was restricted to infected cells. We hypothesized that the parasite induced the expression of this gene to increase proliferation and viability of macrophages [66,67], ensuring therefore its dissemination.

A functional enrichment analysis revealed that *L*. *infantum* infection triggered few significant GO terms. In addition, this parasite was not able to stimulate the inflammasome signaling pathway. Some post-infection up-regulated processes such as *nerve growth factor receptor pathway*, *response to peptide hormone* and *regulation of MAPK cascade* are related to cell signaling and could, theoretically, being activated to sustain host cell survival. Otherwise, the term *negative regulation of transcription from RNA polymerase II* which may be related to gene regulation and homeostasis [68]. Interestingly, some of the up-regulated biological processes seem to be somehow involved in *L*. *infantum* survival. For example, platelet activation is a *child* GO term that was upregulated in *L*. *infantum* infected cells. This finding is in agreement with a study that showed that *Leishmania major* infection activated this pathway and was able to recruit monocytes into lesion and efficiently destroy the parasites [69]. CD36 was one of the up-regulated genes that participates in platelet activation, hemostasis and coagulation pathways. This membrane glycoprotein has been associated to *L*. *amazonensis* and *L*. *major* infection; these parasite species engage host CD36 ligands triggering fusion with late endosomal vesicles and therefore increasing the parasitophorus vacuole size, allowing the acquisition of nutrients, survival, parasite replication and also escape from the immune response [10]. *L*. *donovani* also survives inside macrophages by delaying the fusion of phagosomes with lysosomes [70,71].

Another biological process enriched after *L*. *infantum* infection was the MAPK regulation that includes increased expression of *DUSP2*, *DUSP5* and *DUSP7*, a class of protein phosphatases. DUSPs are expressed during infection with several *Leishmania* species and are able to downregulate the MAPK pathways through p38, JNK, ERK1/2 and PKC dephosphorization. This results in decreased expression of iNOS and other host protective mediators [72–74]. Thus, the over-expression of these pathways suggests that this could be other mechanisms employed by this *Leishmania* specie to disable pro-inflammatory host-cell pathways. In addition, our study showed that infection with *L*. *infantum* did not determine a significant difference in the production of TNF-α and IL-6. This fact is consistent with other studies that showed that *Leishmania* parasites hinder the ERK and p38 phosphorylation and activate protein tyrosine phosphatases, decreasing the production of those cytokines and therefore suppressing the inflammatory response [75–77]. The RPS6KA1, one of most up-regulated gene, also participates in MAPK signaling pathway, downregulating TLR signaling pathway [78–80]. Although we did not found studies that could elucidate the involvement of S6K1 in *Leishmania* infections, we believe that increased expression of this gene may contribute to inactivation of host pro-inflammatory pathways.

The inflammasomes play crucial roles as innate immune sensors [18,81] and many studies support their activation during *Leishmania* infection [31,32,82]. However, the exact role of this cytosolic multiprotein complex in visceral leishmaniasis is not clear yet and emphasis to the inflammasome genes expression by using RNA-seq technique was given in this study. Thus, this is the first study that evaluates the NLR signaling pathway after infection of a human macrophage-like line with *L*. *infantum*. As expected, LPS stimulation elicited the functional enrichment analysis of NLRs signaling pathway available in KEGG database. Otherwise, this pathway was not significantly enriched after infection with *L*. *infantum*. Comparing infected cells with LPS-stimulated cells, the NLRs signaling pathway was one of the biological processes significantly enriched in down-regulated genes. This result shows that *L*. *infantum* was not able to activate inflammasomes in the early stages of infection in this *in vitro* model. In addition to non-expression of inflammasome genes after 8 hours of infection, *L*. *infantum* neither activate caspase-1 nor determined IL-1β production in the three evaluated time-points, confirming that this platform was not indeed activated.

It is well established that *Leishmania* modulates the host immune response and abrogates microbicide mediators to survive and multiply inside host cells [Reviewed in Ref. [2]]. Thus, we interpreted this non-activation of inflammasomes as a possible mechanism used by *Leishmania* to escape from host immunity as already described in the literature [11,33,34]. The first step of NLRP3 activation occurs through the interaction of TLRs, NLRs and cytokine receptors with ligands. This interaction results in NF-kB translocation and the subsequent expression of NLRP3 sensor, pro-caspase-1 and pro-IL-1β [22,83]. However, our results indicate that *L*. *infantum* was not able to increase the expression of NF-κB. This finding is supported by other studies that showed that different *Leishmania* species were able to inactivate this transcription factor through the action of the metalloprotease GP63, which impaired macrophage function and the production of ROS and other inflammatory mediators [77,84]. The relevance of ROS generation as a secondary signal during inflammasome assembly [27] was supported by an investigation employing *L*. *major and L*. *mexicana*. GP63 glycoprotein derived from both species interfered in phosphorylation of PKC signaling pathway, impairing ROS generation and IL-1β production, blocking inflammasome activation [33]. Lipophosphoglycan (LPG), which is present in the *Leishmania* cell membrane, is also able to dysregulate m-RNA IL-1β transcription, compromising its stability and reducing its production [85,86].

In our study, one of the few inflammasome pathway genes that was up regulated by *L*. *infantum* infection was TNFAIP3. This is an interesting finding because this gene encodes the so-called A20 protein which is a prototypical pro-inflammatory mediator in signaling pathways [87,88]. A similar result was found after *L*. *donovani* infection; A20 negatively regulated NF-κB signaling and interfered in NLRP3 inflammasome activation [34]. These results were confirmed by silencing the A20 protein which increased transcription of inflammasome components and maturation of IL-1β, necessary for parasite clearance [34]. Guanylate-binding protein 1 (GBP1) was among the 30-top down-regulated genes after infection with *L*. *infantum*, which also appears to contribute to the suppression of inflammasome activation. This enzyme is known by its relevance in host protection by stimulating both, oxidative killing and transfer of antimicrobial peptides to autophagolysosomes [89]. The GBPs are also involved in the inflammasome and caspase-1 activation through rupture of vacuoles and subsequent release of PAMPs into the cytoplasm [90,91]. Differently from our findings, studies with *Leishmania* have more commonly showed increased GBPs expression upon infection [92,93].

Activation of inflammasomes in *Leishmania* infections is still a very controversial subject. Researchers have shown that infections with species causing cutaneous leishmaniasis result in activation of NLRP3 inflammasomes, which plays a crucial role in host protection and clearance of the parasites [32,94,95]. In this sense, Lima-Junior et al [32] showed that infection with *L*. *amazonensis*, *L*. *braziliensis* and *L*. *major* triggered NLRP3\Caspase-1 activation and IL-1β production. Activation of this complex resulted in increased NO production via NADPH oxidase, restricting intracellular parasite replication *in vivo* and *in vitro* [32]. Similarly, resistance to infection and limitation of *L*. *amazonensis* replication in macrophages resulted from Dectin-1/kinase Syk activation and ROS production, which led to activation of NLRP3 inflammasome [94]. It was also demonstrated that *Leishmania* LPG released into the macrophage´s cytosol triggered non-canonical NLRP3 inflammasome activation through caspase-11 activation [95].

While these studies have shown that inflammasome activation and IL-1β production can control the infection, other authors showed that inflammasome activation can contribute to increased pathology and disease exacerbation [82,96–98]. For example, experimental infection with *L*. *major* resulted in nonhealing cutaneous lesions being this fact related to increased IL-1β production via NLRP3 inflammasome and local neutrophil recruitment [82]. IL-18 production by NLRP3 inflammasome in *L*. *major* infected BALB/c mice determined enhanced IL-4 levels, also allowing parasite´s persistence. NLRP3^-/-^, ASC^-/-^, casp1^-/-^ mice and IL-18 neutralization all conferred greater resistance to infection by decreasing IL-1β and IL-18 production and by controlling parasitic growth [96]. Novais et al. [99] also described upregulation of the NLRP3 pathway in human *L*. *braziliensis* lesions in comparison to samples from healthy subjects [99]. More recently, this research group reported that exacerbation of inflammatory lesions caused by *L*. *major* was due to CD8^+^T cells cytotoxicity, which induced apoptosis of target cells, releasing DAMPs and then activating the inflammasomes [98]. An interesting study done with monocytes from cutaneous leishmaniasis patients revealed NLRP3 inflammasome activation and IL-1β production after stimulation with *L*. *braziliensis* antigens. Area of necrosis and lesion´s permanence were, interestingly, directly related to IL-1β levels [97]. As a whole, these reports indicate that some cutaneous *Leishmania* species determine skin lesions worsening by activating the inflammasome complex.

Similar to our results, elegant studies with viscerotropics species such as *L*. *donovani* has shown that parasite appear to dysregulate inflammasome assembly [34,100]. Gupta et al. [34] revealed that experimental *L*. *donovani* infection culminates in increased A20 expression, determining regulation of NF-kB signaling and non-activation of NLRP3, caspase-1 and pro-IL1-β. Also, ROS generation was regulated by increased mitochondrial uncoupling protein 2 (UCP2) expression [34]. THP-1 cells infected with *L*. *donovani* showed low levels of pro-casp1 expression and lack caspase-1 maturation, which impaired IL-18 and IL-1β production and allowed the increased parasite burden [100]. Surprisingly, researchers have shown that inhibition of inflammasome was also performed by species as *L*. *major*, *L*. *mexicana* and *L*. *guayanenisis*, which inhibited ROS and IL-1β production in THP-1 cells and upregulated A20 expression, respectively [33,35].

Different facts could explain these discordant findings concerning inflammasome activation by *Leishmania*. Methodological details as employed target cells, mice background, timepoint of experimental infections, parasite specie and host genetic factors could all contribute to the controversy that surrounds the induction of inflammasome activation by distinct *Leishmania* species.

In this study we showed, in the early stages of *L*. *infantum* infection, that there is a very low activation of inflammatory pathways, including the NLR signaling pathway that is usually activated during infections caused by cutaneous *Leishmania* species. In addition to non-expression of the inflammasome pathway signaling, *L*. *infantum* was also unable to activate caspase-1 and IL-1β production in early and later periods post-infection. However, GO enriched analysis indicated that a few biological processes were triggered by *L*. *infantum* infection, suggesting therefore that the entrance of this parasite in the target cell is not completely silent. The possible involvement of some of these biological processes in parasite survival deserves further investigation. Our findings, analyzed in the context of the literature lead us to propose that visceral disease causing leishmaniasis, unlike the cutaneous species, use this strategy to facilitate their survival inside host cells.

## Supporting information

S1 File Amastigotes of *L*. *infantum* inside THP-1 cells after 8 hours of infection (1 cell: 10 parasites). The images are representative of at least 3 independent experiments. Slides were added in the wells prior to cells plating. After 8 hours of infection the slides were stained with Giemsa and observed using light microscopy. The top panels represent 400x magnification and the lower ones 1000x magnification.
(TIF)

S2 File Number of reads per sample and percentages of mapped reads over human and *L*. *infantum* genomes. (DOCX)

S3 File Functional enrichment analysis of LPS-stimulated cells in relation to unstimulated cells. Gene Ontology (GO) enrichment analysis summarized by REVIGO showing 40 biological process enriched (*parents* GO terms) in up-regulated genes (FDR<0.01) in cells stimulated by LPS after 8 hours. Highly similar GO terms are linked by edges, where the line width indicates the degree of similarity.
(TIF)

S4 File NLRs signaling pathway. The NOD-like pathway signaling present in the KEGG database was enriched by R pathview package (FDR<0.01) in up-regulated genes of LPS-stimulated cells vs. unstimulated cells. The expression is based on Log2 fold-change <0. Genes with fold-change above 0 are up-regulated (red) and genes with fold-change below 0 are down-regulated (green). The bars indicate the Log2 fold-change.
(TIF)
