# Supplementary figures and images for: Transcriptional analysis of THP-1 cells infected with Leishmania infantum indicates no activation of the inflammasome platform
Source: PLoS Negl Trop Dis. 2020 Jan 21;14(1):e0007949. doi: 10.1371/journal.pntd.0007949 (PMC6994165; doi:10.1371/journal.pntd.0007949)

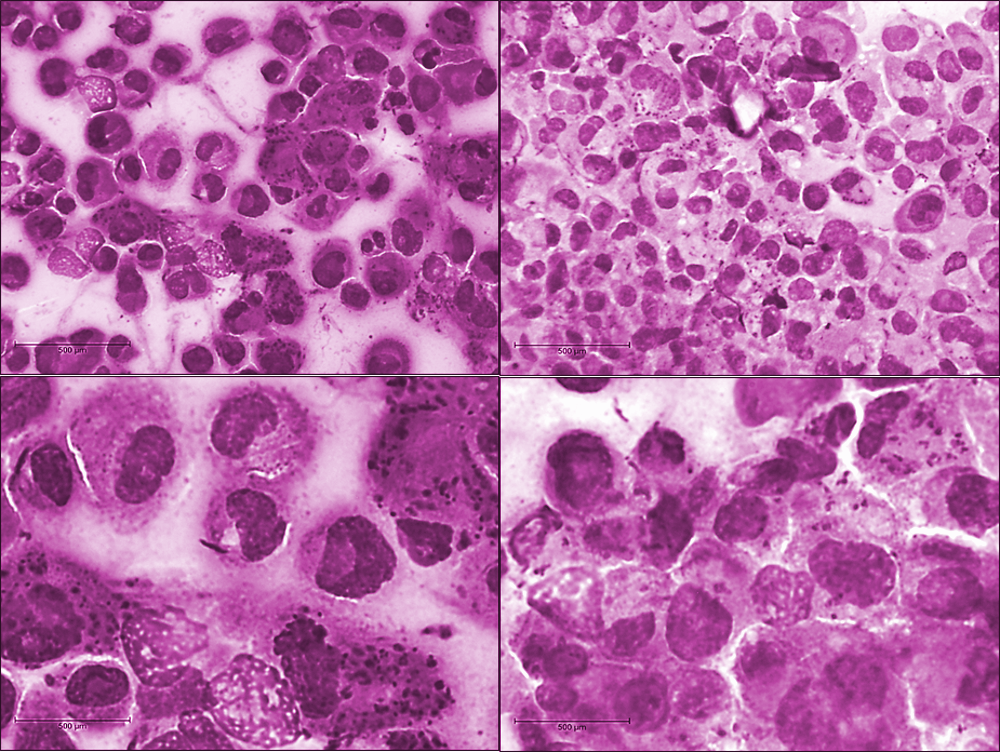

Supplement: S1 File — The images are representative of at least 3 independent experiments. Slides were added in the wells prior to cells plating. After 8 hours of infection the slides were stained with Giemsa and observed using light microscopy. The top panels represent 400x magnification and the lower ones 1000x magnification. (TIF) [file pntd.0007949.s001.tif]

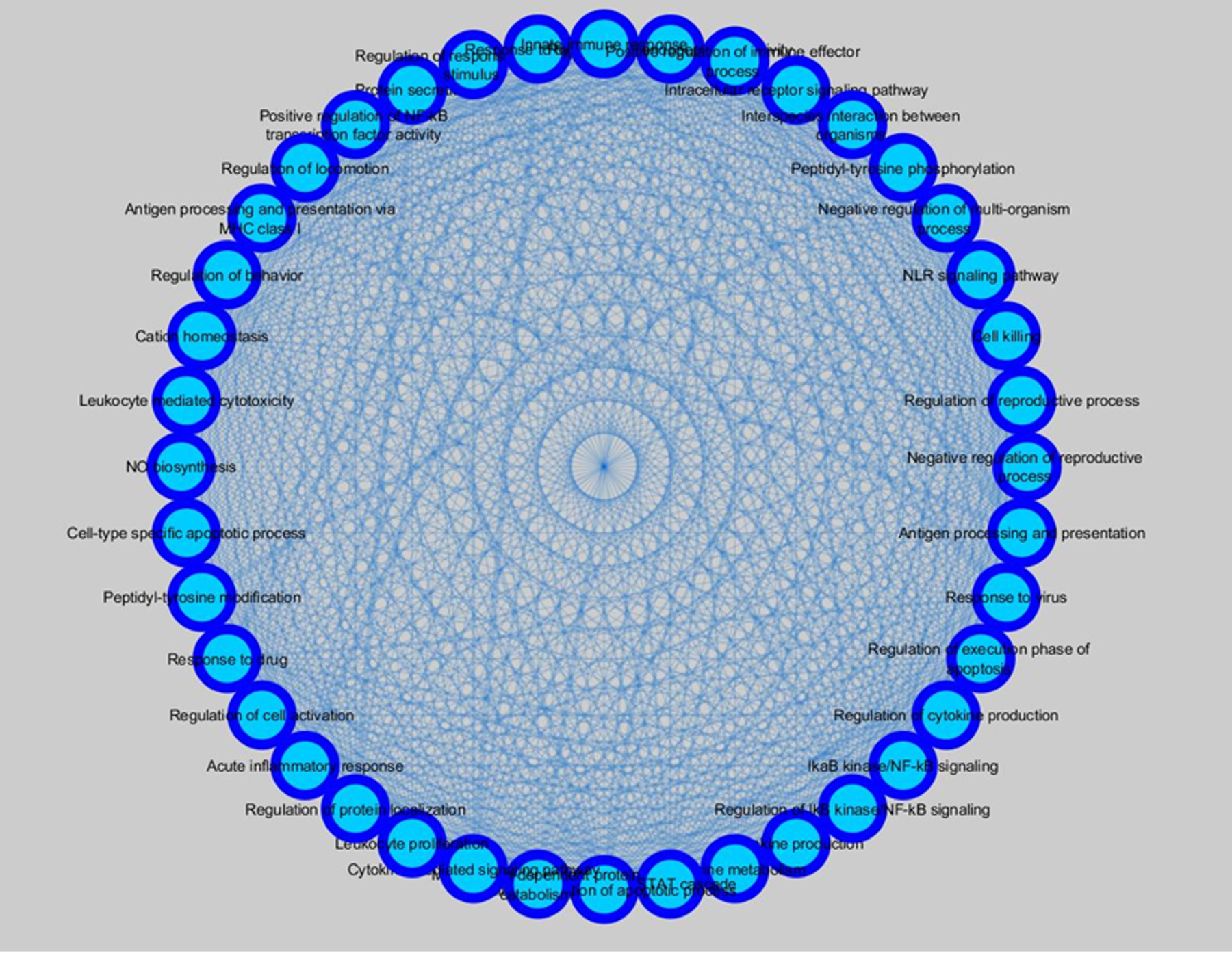

Supplement: S3 File — Gene Ontology (GO) enrichment analysis summarized by REVIGO showing 40 biological process enriched (parents GO terms) in up-regulated genes (FDR<0.01) in cells stimulated by LPS after 8 hours. Highly similar GO terms are linked by edges, where the line width indicates the degree of similarity. (TIF) [file pntd.0007949.s003.tif]

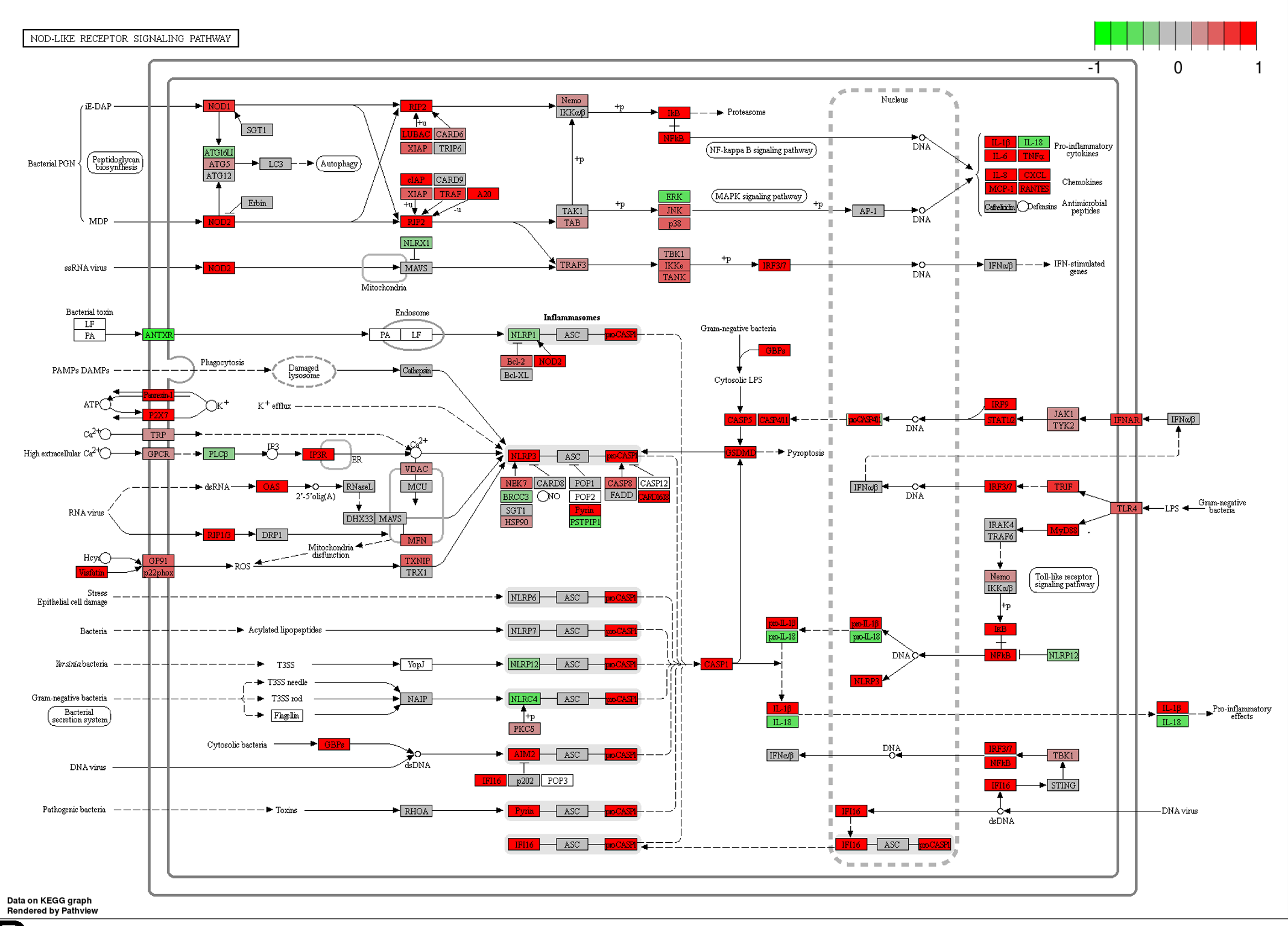

Supplement: S4 File — The NOD-like pathway signaling present in the KEGG database was enriched by R pathview package (FDR<0.01) in up-regulated genes of LPS-stimulated cells vs. unstimulated cells. The expression is based on Log2 fold-change <0. Genes with fold-change above 0 are up-regulated (red) and genes with fold-change below 0 are down-regulated (green). The bars indicate the Log2 fold-change. (TIF) [file pntd.0007949.s004.tif]
