# Supplementary material for: Transcriptional analysis of THP-1 cells infected with Leishmania infantum indicates no activation of the inflammasome platform
Source: PLoS Negl Trop Dis. 2020 Jan 21;14(1):e0007949. doi: 10.1371/journal.pntd.0007949 (PMC6994165; doi:10.1371/journal.pntd.0007949)
Supplement: S2 File — (DOCX) [file pntd.0007949.s002.docx]

|  |  |  |  | **% Aligned reads** | |
| --- | --- | --- | --- | --- | --- |
| **Library** | **Raw data** | **Nº reads after cleaning** | **SLAC** | ***Homo sapiens*** | ***L. infantum*** |
| **M1** | 15700706 | 10.371.565,00 | 60-76 | 97,97 | 0 |
| **M2** | 15957144 | 10.472.486,00 | 60-76 | 97,62 | 0 |
| **M3** | 72441126 | 46.947.117,00 | 60-76 | 97,62 | 0 |
| **M4** | 16717649 | 10.772.089,00 | 60-76 | 98,06 | 0 |
| **M5** | 17903053 | 11.778.327,00 | 60-76 | 98,13 | 0 |
| **M6** | 14868725 | 9.365.537,00 | 60-76 | 98,02 | 0 |
| **M7** | 17751944 | 11.760.784,00 | 60-76 | 96,77 | 1,37 |
| **M8** | 13878386 | 9.214.425,00 | 60-76 | 96,72 | 1,29 |
| **M9** | 16581684 | 10.893.453,00 | 60-76 | 96,65 | 1,48 |

SLAC: Sequence length after cleaning

**Supplementary File 3**. Top 30 up and down-regulated genes in THP-1 infected with *Leishmania*

*infantum* in relation to unstimulated cells.

| **UP-REGULATED GENES** | | |
| --- | --- | --- |
| **Gene** | **p-value** | **FC (fold-change)** |
| **AGRN** - Agrin | 3.10E-05 | 0.22 |
| **ENO1** – Enolase1 | 5.52E-06 | 0.23 |
| **TNFRSF14** - TNF receptor superfamily member 14 | 9.43E-05 | 0.39 |
| **KLHL21** - Kelch like family member 21 | 9.93E-06 | 0.41 |
| **PLEKHM2** - Pleckstrin homology and RUN domain containing M2 | 3.18E-06 | 0.22 |
| **AL450998.2** – *Novel transcript* | 1.21E-05 | 0.22 |
| **CCNL2** - Cyclin L2 | 5.21E-06 | 0.28 |
| **RIT1** - Ras like without CAAX 1 | 2.98E-08 | 0.32 |
| **ZBTB7B** - Zinc finger and BTB domain containing 7B | 1.78E-06 | 0.30 |
| **CDC20** - Cell division cycle 20 | 1.26E-08 | 0.31 |
| **SLC16A1** - Solute carrier family 16 member 1 | 1.89E-04 | 0.23 |
| **LMO4** - LIM domain only 4 | 2.30E-17 | 0.33 |
| **RPS6KA1** - Ribosomal protein S6 kinase A1 | 1.66E-05 | 0.14 |
| **GFI1** - Growth factor independent 1 transcriptional repressor | 4.24E-07 | 0.39 |
| **KIF1B** - Kinesin family member 1B | 4.57E-04 | 0.20 |
| **CDKN2C** - Cyclin dependent kinase inhibitor 2C | 1.82E-05 | 0.33 |
| **NOTCH2** - Notch 2 | 2.00E-04 | 0.17 |
| **THEMIS2** - Thymocyte selection associated family member 2 | 1.59E-04 | 0.25 |
| **KIAA1522** – Human gene* | 5.75E-12 | 0.45 |
| **IL6R** - Interleukin 6 receptor | 1.78E-09 | 0.27 |
| **AL390719.1** – Protein tyrosine phosphatase family pseudogene | 3.95E-04 | 0.29 |
| **SELENON** - Selenoprotein N | 2.15E-04 | 0.15 |
| **SPSB1** - SplA/ryanodine receptor domain and SOCS box containing 1 | 4.68E-09 | 0.34 |
| **SFPQ** - Splicing factor proline and glutamine rich | 1.19E-04 | 0.14 |
| **HSPB7** - Heat shock protein family B (small) member 7 | 5.79E-07 | 0.22 |
| **PLEKOH1** - Pleckstrin homology domain containing O1 | 1.05E-06 | 0.21 |
| **TNFRSF1B** - TNF receptor superfamily member 1B | 2.72E-24 | 0.40 |
| **CSF1** - Colony stimulating factor 1 | 5.50E-19 | 0.52 |
| **SHE** - Src homology 2 domain containing E | 9.69E-05 | 0.60 |
| **MTX1P1** - Metaxin 1 pseudogene 1 | 8.99E-05 | 0.31 |
| **DOWN-REGULATED GENES** | | |
| **Gene** | **p-value** | **FC (fold-change)** |
| **SF3A3** – Splicing factor 3a subunit 3 | 2.55E-04 | -0.23 |
| **WDR3** - WD repeat domain 3 | 8.49E-06 | -0.37 |
| **GBP1** - Guanylate binding protein 1 | 5.56E-07 | -0.61 |
| **LBH** - Limb bud and heart development | 1.40E-10 | -0.49 |
| **TMEM43** - Transmembrane protein 43 | 1.58E-06 | -0.27 |
| **AC090004.1** – *Novel transcript* | 9.57E-05 | -0.24 |
| **DDX18** - DEAD-box helicase 18 | 6.06E-05 | -0.27 |
| **ACKR3** - Atypical chemokine receptor 3 | 4.11E-04 | -0.49 |
| **MYCBP** - MYC binding protein | 1.69E-04 | -0.41 |
| **AL365181.3** – *Novel transcript*, antisense to BCAN | 2.25E-07 | -0.69 |
| **AL139260.1** – *Novel transcript* | 1.76E-04 | -0.38 |
| **BCAN** – Brevican | 1.37E-11 | -0.70 |
| **ANTXR1** - Anthrax toxin receptor 1 | 3.02E-04 | -0.50 |
| **BTF3L4** - Basic transcription factor 3 like 4 | 8.44E-05 | -0.28 |
| **ALG1L6P** - Asparagine-linked glycosylation 1-like 6, pseudogene | 5.55E-04 | -0.53 |
| **AL360270.1** –  *Novel transcript* | 1.04e-04 | -0.35 |
| **C2orf88** - Chromosome 2 open reading frame 88 | 7.76e-05 | -0.26 |
| **F3** - Coagulation factor III, tissue factor | 4.04E-08 | -0.47 |
| **RPS24P8** - Ribosomal protein S24 pseudogene 8 | 2.35E-04 | -0.23 |
| **FLVCR1** - Feline leukemia virus subgroup C cellular receptor 1 | 5.05E-05 | -0.65 |
| **UCK2** - Uridine-cytidine kinase 2 | 2.85E-04 | -0.30 |
| **FAM86DP** - Family sequence similarity 86 member D, pseudogene | 3.37E-04 | -0.41 |
| **ARID4B** - AT-rich interaction domain 4B | 7.45E-05 | -0.22 |
| **HSD3BP5** - Hydroxy-delta-5-steroid dehydrogenase,3 beta , pseudogene 5 | 5.78E-06 | -0.59 |
| **HNRNPR** - Heterogeneous nuclear ribonucleoprotein R | 1.74E-07 | -0.31 |
| **LRIF1** - Ligand dependent nuclear receptor interacting factor 1 | 3.76E-04 | -0.30 |
| **MTCO3P12** - Mitochondrially encoded cytochrome c oxidase III pseudogene 12 | 3.57E-07 | -0.32 |
| **MTCO1P12** - Mitochondrially encoded cytochrome c oxidase I pseudogene 12 | 1.71E-09 | -0.29 |
| **MTND1P23** - Mitochondrially encoded NADH: ubiquinone oxidoreductase core subunit 1 pseudogene 23 | 5.66E-06 | -0.45 |
| **EEF1B2** - Eukaryotic translation elongation factor 1 beta 2 | 1.64E-04 | -0.17 |
